# Sustainable and accessible hemodialysis: life cycle assessment on central acid delivery system

**DOI:** 10.1186/s12882-025-04499-0

**Published:** 2025-11-05

**Authors:** Chang-Lung Tsai, Abass Fehintola, Guus Crooijmans, Jeroen Vollenbroek, Brett Duane, Karin Gerritsen

**Affiliations:** 1 Department of Nephrology and Hypertension, University Medical Center Utrecht, Heidelberglaan 100, Utrecht, 3584 CX The Netherlands; 2 School of Dental Science, Trinity College Dublin, Dublin, Ireland; 3 BIOS Lab on a Chip Group, University of Twente, Enschede, The Netherlands

**Keywords:** Hemodialysis, Green nephrology, Sustainability, Global health, Central concentrate delivery system

## Abstract

**Introduction:**

The healthcare sector contributes over 4% of global greenhouse gas emissions. As healthcare systems pursue carbon reduction targets, sustainable innovations in clinical operations are essential. Hemodialysis (HD) relies heavily on dialysate concentrates as one of its major consumables. This study evaluates the environmental impact of implementing a central acid concentrate delivery system (CCDS) using dry concentrate powder at the University Medical Center Utrecht (UMC Utrecht) and examines its broader global applicability.

**Methods:**

A comparative life cycle assessment (LCA) was conducted to assess the environmental impacts of acid concentrate delivery via single-use liquid canisters versus CCDS using dry powder in reusable barrels at UMC Utrecht. The analysis includes additional scenarios involving long-distance distribution to Italy in Europe, Kenya in Africa, and the Philippines in Asia.

**Results:**

Implementation of CCDS at UMC Utrecht reduced carbon emissions by 58%. Endpoint impacts on human health, ecosystem quality, and resource scarcity were reduced by more than half. Despite longer transport distances in global scenarios, CCDS consistently outperformed the canister system across most impact categories, driven by reuse of packaging and reduced transport volume and weight.

**Conclusions:**

CCDS offers a more sustainable alternative, with substantial environmental benefits even in long-distance applications. Operational advantages include reduced manual handling and simplified logistics. By enabling local dialysate production and decreasing reliance on imported disposables, CCDS may also improve accessibility in low-resource settings. Widespread adoption could thereby advance environmental sustainability globally.

**Supplementary Information:**

The online version contains supplementary material available at 10.1186/s12882-025-04499-0.

## Introduction

The healthcare sector is a major contributor to global greenhouse gas emissions, responsible for more than 4% of total emissions and up to 10% in some high-income countries [1, 2]. In the Netherlands, pharmaceuticals and chemical products are the leading sources of healthcare-related environmental impacts, contributing 41% to the sector’s climate change footprint [3]. To align with net-zero targets by 2050 [4–6], the European Green Deal aims to reduce emissions by 55% by 2030 [7].

Chronic kidney disease (CKD) represents a growing burden within healthcare, affecting approximately 850 million people globally [8], with nearly 5 million requiring kidney replacement therapy (KRT) [9]. Of these, around 70% receive in-center hemodialysis (HD) [10]. Prevalence is especially high in high-income countries [11], while in many low- and middle-income countries (LMICs) HD is often the only option due to limited transplant infrastructure, financial barriers, and constrained healthcare capacity [12]. A single HD patient can produce up to 10 tons of carbon emissions annually [13, 14], far exceeding the estimated 150 kg associated with a standard surgery [15]. Reducing the environmental footprint of dialysis is therefore essential to break the vicious cycle between clinical and environmental burden [16, 17].

The majority of sustainability challenges within hospitals is largely associated with the linear supply chain model, dominated by procurement and use of single-use consumables [18]. Embedding circular economy strategies, such as refuse, reduce, reuse, and the broader 10R framework, offer opportunities to minimize resource use, avoid waste, and retain material value across the product life cycle [19, 20].

This study focuses on innovations in dialysate delivery, the largest consumable in HD, specifically the adoption of a central concentrate delivery systems (CCDS). At the University Medical Center Utrecht (UMC Utrecht), despite being a relatively low-volume dialysis center, consumes approximately 40,000 L of acid concentrate annually, resulting in the disposal of around 5,000 single-use acid concentrate canisters. In 2022 the center introduced a CCDS, the EcoMix Revolution (B. Braun, Germany) [21]. Although centralized systems have long been established in countries such as Japan [22], they remain a minority in Europe and North America, where canister systems dominate.

**Fig. 1:**
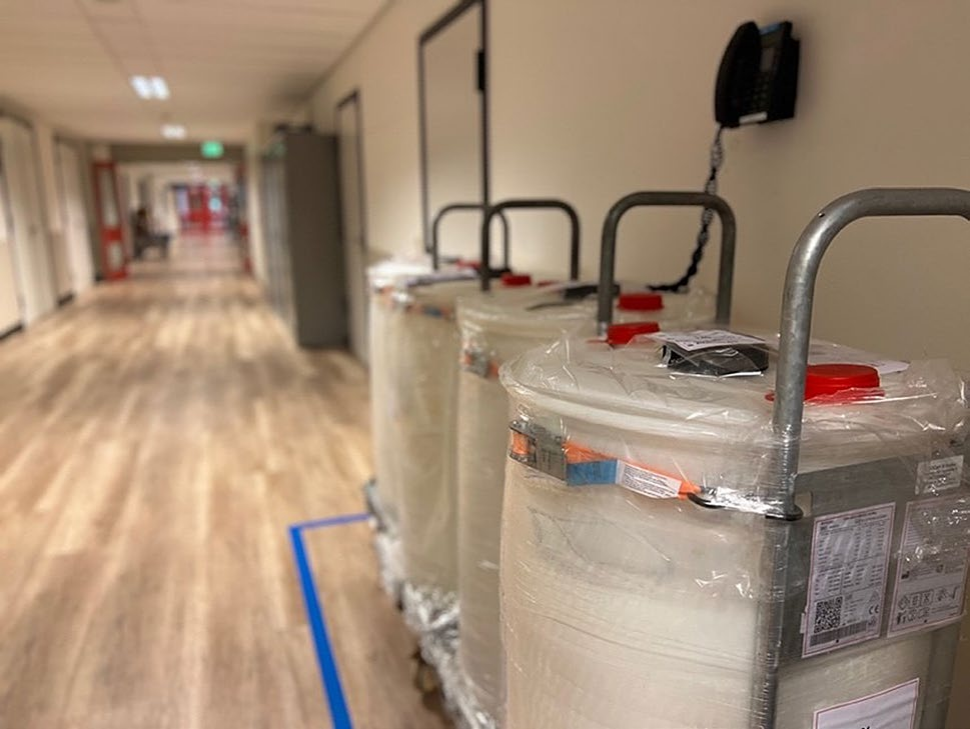
Barrels of dry acid concentrates transported to the dialysis unit at UMC Utrecht. (Source: Authors)

The system at UMC Utrecht uses reusable EcoCart barrels [23] (Fig. 1), each containing 216.4 kg of powder to produce 622 L of acid concentrate. Bulk delivery of the dry concentrate reduces transportation load and plastic wastes. The solution is stored in 860-liter tanks and distributed to dialysis machines via a central loop, which requires less attention and labor from healthcare personnel. Table 1 compares consumables used in CCDS and conventional canister system previously used in UMC Utrecht.

Previous studies have demonstrated reductions in packaging waste and residual chemicals with central delivery systems [24, 25], but a comprehensive life cycle assessment (LCA) has not been performed. LCA is increasingly recognized as a powerful decision-support tool in hospitals, helping managers evaluate alternatives and identify environmental hotspots [26]. This study addresses that gap by quantifying the carbon emission reductions achieved at UMC Utrecht. It further evaluates whether CCDS remains advantageous when shipped over longer distances, compared with locally sourced canister systems. The findings aim to support evidence-based decisions for healthcare providers and highlight how innovative sustainable technologies could help strengthen dialysis capacity and resilience in LMICs.

**Table 1:** Comparison of canister and central concentrate delivery systems. (Illustration generated with openai based on author-provided specifications)

System	Canister System	Central Concentrate Delivery System
Reference Model	Dirinco 831	B. Braun EcoCart
Use Cycle	Disposable canister	Reusable barrel (15 times)
Transport carrier	Wooden Pallet	Steel Cart
Capacity	6 L	216.4 kg (equivalent to 622 L of concentrate)
Production Location	Neubrandenburg, Germany	Ostrhauderfehn, Germany
Transport Distance	710 km	245 km
Image		

## Materials and methods

An LCA evaluates the environmental impacts of a product across all stages of its life, including raw material extraction, production, distribution, use, to final disposal. This study followed the ISO 14040 framework for life cycle assessment (LCA), which consists of four iterative phases: (1) goal and scope definition, (2) life cycle inventory analysis, (3) life cycle impact assessment, and (4) interpretation [27]. This involves first defining the problem and the purpose of the study (goal and scope), then collecting the relevant data (inventory analysis). Next, the data are entered into the model and the environmental impacts are calculated (impact assessment), and finally, the results are analyzed and explained in context (interpretation). The following sections describe how each of these phases was applied to the comparison of the canister and central concentrate delivery systems.

### Goal and scope definition

The objective of this study is to estimate and compare the environmental impact of two delivery models for acid concentrate used in HD: the linear single-use acid concentrate system with disposable canisters and the circular central delivery system utilizing dry powdered acid concentrates in reusable barrels.

The functional unit is defined as the acid concentrate needed to supply one HD machine for one patient (3× weekly, 4 h) for one year. Each session requires approximately 4 L of 44:1 dilution acid concentrate using a standard dialysate flow of 500 mL/min, resulting in an annual requirement of 624 L per patient.

The system boundaries include the production of acid concentrate, either in liquid or powdered form, the packaging of the product (disposable canisters or reusable barrels), the transportation of the concentrate to the hospital, and the end-of-life treatment of both the acid concentrate and the associated packaging materials. Capital goods, such as machines, process equipment, and building infrastructure, are excluded from the assessment due to their extended lifespans and marginal influence within the defined functional unit [27].

**Fig. 2:**
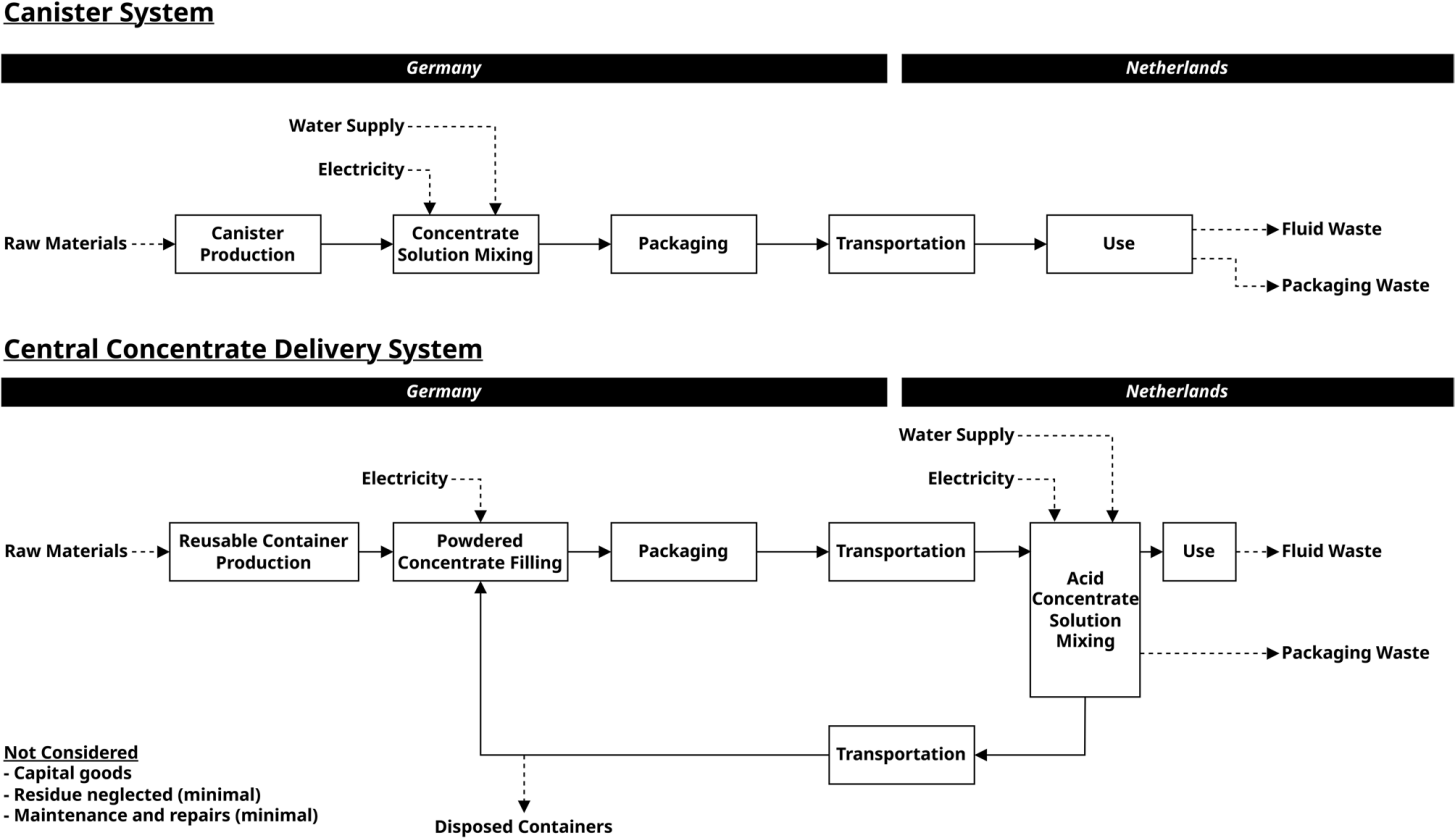
Process flow diagram of the delivery of acid concentrates. The upper diagram shows a single-use system using canisters; the lower diagram shows a central concentrate delivery system using dry concentrate powder and reusable barrels. Capital goods are excluded from the analysis. Residues and maintenance are not considered due to their minimal presence in the system

The process flows for canister and central delivery systems are illustrated in Fig. 2. In the canister system, acid concentrate is produced in liquid form and packed in 6-liter canisters at the manufacturing site, and transported to the hospital. At UMC Utrecht, canisters are used for more than one patient until it is (almost) empty, meaning no liquid is discarded and only minimal residue remains. The empty canisters are discarded after single use.

In the centralized system, acid concentrate is initially produced as a dry powder. It is then transported to the hospital, where it is dissolved and mixed with RO water to produce the concentrate solution used for dialysis. The reusable barrels are returned to the manufacturer and reused for a minimum of 15 cycles, based on conservative estimates provided by the supplier.

To explore the influence of geographic distance, additional scenarios were modeled to represent different delivery locations, including a more distant European site and two LMICs in Africa and Southeast Asia, where expanding access to HD is particularly relevant. These sites include:

- Europe: University of Modena and Reggio Emilia (Italy),
- Africa: Kenyatta National Hospital (Nairobi, Kenya),
- Asia: Philippine General Hospital (Manila, Philippines).

### Inventory analysis

Data for the inventory analysis were collected between January and April 2025. Primary data such as direct measurement, product documents, and supplier information were used when available. Where such data were unavailable or incomplete, secondary data was used from the Ecoinvent database (version 3.9.1) [28]. A complete description of the life cycle inventory data, assumptions, and calculation parameters is provided in Supplementary Material 1 (Supplementary Methods: Inventory Analysis).

### Impact assessment

The life cycle impact assessment (LCIA) was conducted using the ReCiPe 2016 Midpoint (Hierarchist) method [29]. This perspective was chosen as it reflects a balanced, consensus-based approach, aligned with widely accepted policies and scientific agreements, applying a 100 year time horizon for assessing environmental impacts. Compared to the Individualist (short term) and Egalitarian (long term, precautionary) perspectives, the Hierarchist represents the most commonly used and policy aligned framework.

ReCiPe 2016 Midpoint (Hierarchist) provides characterization across seventeen midpoint impact categories, addressing various environmental issues such as climate change, acidification, and eutrophication.

These midpoint categories are further grouped into three broader endpoint damage categories:

- Human Health: measured in disability-adjusted life years (DALYs).
- Ecosystem quality: expressed as species loss over time (species-years).
- Resource scarcity: represented by the additional financial cost of future mineral and fossil resource extraction, measured in USD.

The complete list of midpoint and endpoint impact categories used in this study is provided in Table 2. Calculations in this study were performed using openLCA 2.4.1 (GreenDelta GmbH, Berlin, Germany).

**Table 2:** List of midpoint and endpoint impact categories for recipe 2016 [29]

Impact category	Abbreviation	Reference unit
Acidification: Terrestrial	TA	kg SO_2_-Eq
Global Warming Potential	GWP	kg CO_2_-Eq
Ecotoxicity: Freshwater	FEc	kg 1,4-DCB-Eq
Ecotoxicity: Marine	MEc	kg 1,4-DCB-Eq
Ecotoxicity: Terrestrial	TEc	kg 1,4-DCB-Eq
Fossil Energy Resources	FR	kg oil-Eq
Eutrophication: Freshwater	FEu	kg P-Eq
Eutrophication: Marine	MEu	kg N-Eq
Human toxicity: Carcinogenic	HCT	kg 1,4-DCB-Eq
Human toxicity: Non-Carcinogenic	HNCT	kg 1,4-DCB-Eq
Ionizing Radiation	IR	kBq Co-60-Eq
Land Use	LU	m^2^ a crop-Eq
Mineral Resources	MR	kg Cu-Eq
Ozone Depletion	OD	kg CFC-11-Eq
Particulate Matter Formation	FP	kg PM2.5-Eq
Photochemical Oxidant Formation	OF	kg NO_x_-Eq
Water Consumption	WC	m^3^
Ecosystem Quality	EQ	species.yr
Human Health	HH	DALYs
Resource Scarcity	RS	USD 2013

### Sensitivity analysis

A sensitivity analysis was conducted across different phases of the life cycle to evaluate how key parameters influence the overall impact. The human health endpoint was selected for comparison, reflecting the importance of health outcomes in the context of medical devices. This analysis compared regional delivery of the canister system to the Netherlands with overseas delivery of the central delivery system to Kenya.

In each phase of the life cycle, including production, packaging, transportation, and end-of-life treatment, one variable was adjusted to simulate potential changes. For production, water and energy use during acid concentrate manufacturing was increased by 100%. In the packaging phase, reusable containers in the central system were replaced with single use packaging. For transportation, the delivery distance was extended by 20% to reflect longer or less efficient shipping routes. For end-of-life treatment, plastic waste that was originally assumed to be recycled was instead treated through incineration.

## Results

### Global warming

**Fig. 3:**
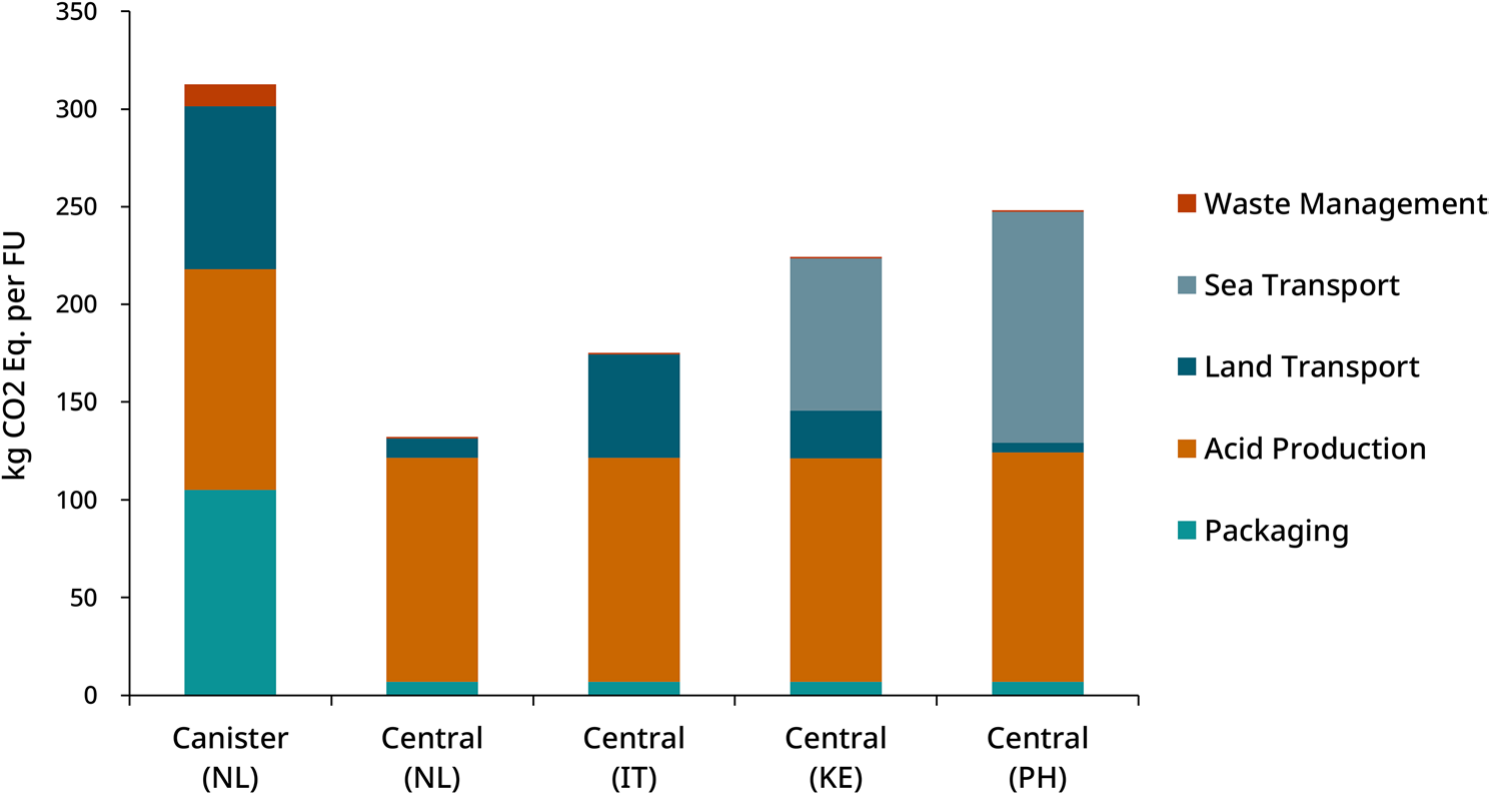
Global warming potential (carbon footprint) of canister and central concentrate delivery system for different scenarios. Emissions are reduced by 58% for deliveries within the Netherlands. The centralized system consistently shows lower emissions due to reduced packaging and transport weight, even in long-distance international shipments

The global warming potential (GWP) of the canister system and central delivery systems is presented in Fig. 3, with detailed values shown in Table S6 (Supplementary Material 2). The central system demonstrates a 58% lower carbon footprint compared to the single-use canister system for deliveries within the Netherlands, mainly attributed to minimized packaging and the efficiency of bulk transport.

Packaging and transport are the main contributors to the GWP of the canister system. Each functional unit requires 28.1 kg of High-Density Polyethylene (HDPE) in the canister system, compared to 1.5 kg in the centralized system, accounting for 15 reuse cycles (Table S5). The mass of transported product per functional unit was reduced from 821 kg in the canister system to 250 kg in the central system (Table S2), contributing to a substantial reduction in transport-related carbon emissions.

Given the proximity of the manufacturing site to the delivery location in the Netherlands, we also assessed transport emissions for alternative, more distant destinations. Transport emissions increase with distance, particularly for sea transport deliveries to Africa and Asia. However, the overall environmental impact of the central system remains substantially lower than that of the canister system.

Emissions from acid production (113 kg CO2-Eq) include raw materials, water, and electricity consumption. A substantial share of these emissions is attributed to glucose use (42% of acid production) and other raw chemicals used in the formulation of the concentrates (together 55%). Water and energy consumption contribute less than 3% of acid production-related emissions.

Finally, waste management, including wastewater treatment and recycling activities, contributes minimally to total emissions, accounting for 3.5% in the canister system and less than 1% in the central delivery system.

### Other midpoint and endpoint estimates

In the Netherlands, to supply a patient acid concentrates annually, the canister system resulted in 7.46E − 04 DALYs, 1.62E − 06 species·yr, and 50 USD for human health, ecosystem quality, and resource scarcity, respectively. The central system consistently outperforms the canister system across all midpoint and endpoint indicators, reducing ecosystem quality impacts to 48%, human health impacts to 49%, and resource scarcity to 39%. With more distant overseas transport, certain midpoint impact categories become more severe, particularly terrestrial acidification (TA), fine particulate formation (FP), ozone depletion (OD), and photochemical oxidant formation (OF). These factors substantially contribute to the endpoint categories of human health and ecosystem quality. While ships are generally more fuel-efficient per ton-kilometer than road freight, they generate considerable emissions due to the long distances involved in overseas transport. Container ships primarily use heavy fuel oil, which is a source of pollutants such as sulfur dioxide (SO₂), nitrogen oxides (NOx), and fine particulate matter (PM₂.₅) [30].

In contrast, impact on resource scarcity remains relatively low for the central delivery system, also in long-distance transport scenarios, primarily due to the reduced use of packaging materials.

**Fig. 4:**
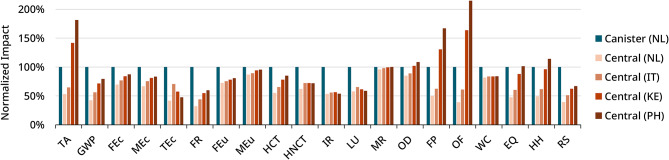
Normalized ReCiPe midpoint and endpoint indicators for canister and central delivery systems across scenarios, with the canister system set as the reference (100%). The central system shows lower impacts overall, though some indicators (TA, FP, OF) increase in long-distance deliveries to Kenya and the Philippines (TA = Acidification: Terrestrial; GWP = Global Warming Potential; FEc = Ecotoxicity: Freshwater; MEc = Ecotoxicity: Marine; TEc = Ecotoxicity: Terrestrial; FR = Fossil Energy Resources; FEu = Eutrophication: Freshwater; MEu = Eutrophication: Marine; HCT = Human Toxicity: Carcinogenic; HNCT = Human Toxicity: Non-Carcinogenic; IR = Ionizing Radiation; LU = Land Use; MR = Mineral Resources; OD = Ozone Depletion; FP = Particulate Matter Formation; OF = Photochemical Oxidant Formation; WC = Water Consumption; EQ = Ecosystem Quality; HH = Human Health; RS = Resource Scarcity)

Overview of the normalised indicator results for all ReCiPe midpoint and endpoint indicators for the canister and central delivery system in different scenarios is shown in Fig. 4.

Full calculation results of the midpoint and endpoint impacts are provided in Table S7 (Supplementary Material 2).

### Sensitivity analysis

**Fig. 5:**
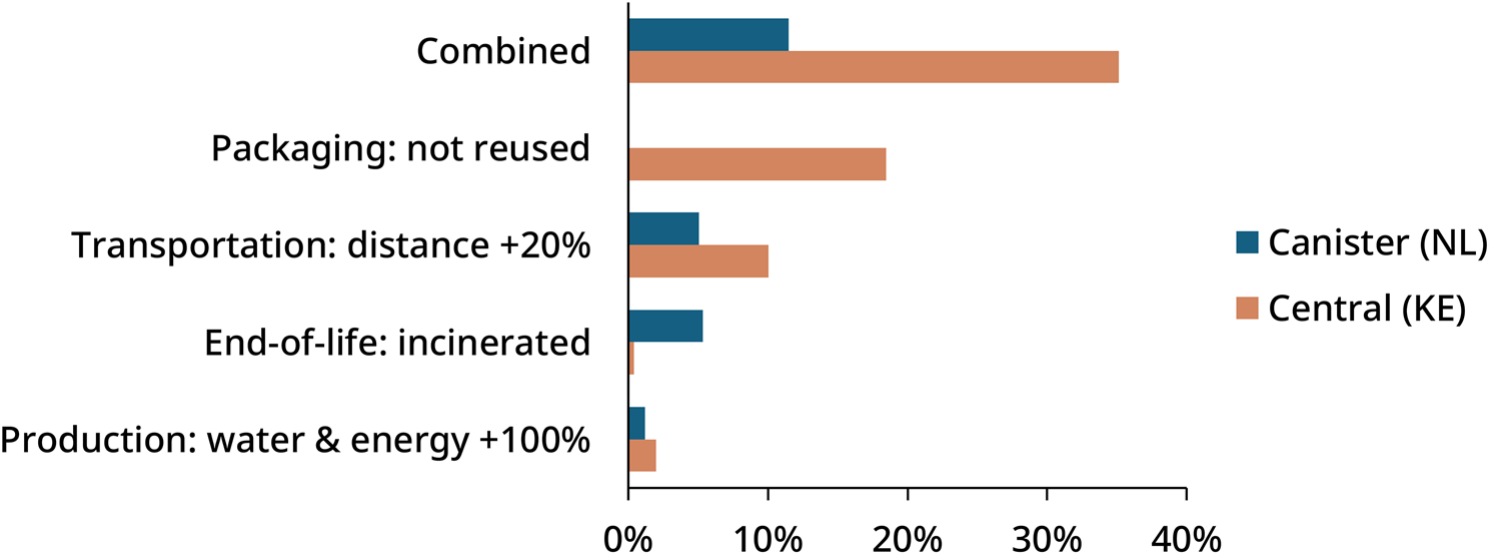
Relative increase of human health impact for canister system (NL) and central delivery system (KE). The central system is more sensitive to packaging reuse and transport distance, due to its larger contribution. In the combined worst-case scenario, the central system shows higher increase of impacts, mainly due to the combination of incinerating additional plastics, highlighting the importance of packaging reuse

Results of the sensitivity analysis, comparing regional delivery of the canister system to the Netherlands with overseas delivery of the central delivery system to Kenya, are presented in Fig. 5. Where parameters are ranked from highest to lowest influence. Detailed values provided in Table S8 (Supplementary Material 2). The central delivery system showed greater sensitivity to changes in packaging and transportation, with impacts increasing by more than 10% in both cases. This highlights the importance of packaging reuse in reducing the overall footprint of centralized systems, and the significant role of transportation due to the longer distances involved. In contrast, the canister system was more sensitive to end-of-life assumptions, largely because it generates more plastic waste. As shown in Table S5, the canister system produces 28.1 kg of plastic waste per functional unit, compared to 1.5 kg for the central system.

Increasing water and energy use during acid production had minimal effect on both systems, confirming that this phase contributes relatively little to the total impact. Most of the environmental burden in acid production comes from the use of raw chemicals rather than utilities.

In the combined worst-case scenario, where all parameter changes were applied at once, the central delivery system showed a significantly higher total impact. This increase was further driven by the combined effects of single-use packaging and the incineration of the additional plastic waste, which together amplified the environmental burden at the end-of-life stage.

## Discussion

### Sustainability performance

This study conducted a full life cycle analysis comparing canister and centralized concentrate delivery systems. At the University Medical Center Utrecht (UMC Utrecht), switching from the canister to central delivery system cut carbon emissions by 58% through reduced transportation and packaging, demonstrating a clear improvement in sustainability. Given the proximity of the manufacturing site in Germany to the delivery location in the Netherlands, additional scenarios involving longer transport distances were modeled to improve generalizability of our findings. Even when delivered to the Philippines, the global warming potential of the central concentrate delivery system remained relatively low, underscoring its environmental advantage. However, at extreme transport distances, some of the benefits of centralized delivery are offset by the increased environmental impacts associated with long-haul shipping. Container ships are major polluters, contributing substantially to acidification and particulate pollution [30]. As a result, when transported over very long distances, the overall endpoint impacts of central delivery may approach those of canister systems delivered over shorter distances for hospitals situated near canister manufacturers.

The sensitivity analysis further underscores the critical role of container reuse. Impacts associated with the production and end-of-life treatment of plastics are substantially reduced through the return and reuse of containers, even when accounting for the additional transportation involved in return shipping over long distances. As shown in Fig. 5, without container reuse would increase the associated impacts for Kenya by nearly 20%, even when return shipping to Europe is excluded. This is because packaging reuse significantly reduces emissions by approximately one order of magnitude, assuming 15 cycles of reuse. In contrast, most transport related emissions originate from outbound shipments due to the greater weight of filled containers, while return trips with empty containers contribute minimally.

### Improved accessibility

Access to dialysis remains critically limited in LMICs, where fewer than 10% of patients with end-stage kidney disease receive treatment due to economic, infrastructural, and logistical barriers [31, 32]. Central delivery systems offer practical advantages by significantly reducing the volume and weight of acid concentrate shipments, which can lower transportation costs and simplify logistics. Moreover, by promoting the local production of dialysates from dry concentrate, these systems can reduce reliance on imported pre-packaged solutions, fostering more resilient and self-sufficient dialysis infrastructure in resource-limited settings.

### Clinical considerations

Beyond environmental gains, central delivery systems also enhance operational efficiency by minimizing manual handling and streamlining concentrate distribution, thereby reducing clinical staff burden and allowing greater focus on patient care. In UMC Utrecht, where acid concentrates are used for more than one patients, residue is minimal. However, in hospitals where residual acid is discarded, these systems provide additional benefit [25]. Maintenance requirements are minimal, with no routine disinfection and only a filter replacement every two years (~ 300 g) [33]. The concentrates have a long shelf life (12 months in dry form and up to 24 months after formulation), allowing for better stock management and reduced risk of supply shortages, especially in remote or high-volume centers [33].

### Limitations

Capital goods such as manufacturing equipment and machinery were excluded due to data limitations and are commonly omitted in assessments in healthcare [34, 35]. However, their impact is expected to be low, given the high production volume at the manufacturing sites in Germany diluting their per-unit impact. Similarly, the centralized system at UMC Utrecht supports over 7,000 dialysis sessions annually and has a lifespan of at least 20 years minimizing its per-unit impact. Literature suggests capital goods typically contribute less than 10% of total environmental impact [27].

Water and electricity use was based on supplier data, with consumption for the canister system estimated as equivalent to the central concentrate system and scaled by weight. As raw chemical inputs drive most production impacts, the influence of water and energy use is minimal on the final results, as confirmed by sensitivity analysis.

End-of-life modeling assumed local recycling and excluded waste transport, negligible compared to the product transport from the manufacturer. While high recycling rates justify this assumption in the Netherlands, incineration was modeled in the sensitivity analysis to reflect regions with limited waste management infrastructure, such as LMICs. It showed only modest increases in impact: 5.3% (canister) and 0.4% (central delivery), suggesting that central delivery systems remain the more sustainable option also in settings with poor waste treatment infrastructure.

### Future research

Implementing central delivery systems would require an additional investment, particularly in Europe, where most dialysis centers are built around canister systems. In contrast, in Japan, where central delivery systems are more widely adopted, both initial and maintenance costs are reported to be lower [22].

To support broader adoption, future research should assess the total cost of ownership, including procurement, labor, maintenance, repair, and end-of-life disposal, and integrate this with environmental analysis through life cycle assessment. Using methods such as environmental pricing [36] and cost-benefit analysis [37] would allow comparison of the social costs of central concentrate delivery and canister systems, supporting better decision making.

Particular attention is needed for LMICs, where healthcare systems may face higher financial and environmental burdens due to longer distances for technical support, limited local infrastructure, and increased vulnerability to system failures [38, 39]. While our analysis indicates that the central delivery system maintains favorable environmental performance even with long-distance transport, broader global implementation, especially LMICs, would likely require more local production of key components such as chemicals. This would reduce dependence on international supply chains and strengthen the long-term feasibility of the system.

While this study highlights the environmental benefits of central delivery systems, further work is needed to develop business models that incentivize adoption in both high- and low-resource settings. This includes financing strategies aligning hospital decision-making with long-term sustainability and ensuring manufacturer profitability in diverse global markets.

## Conclusion

This study demonstrates that transitioning from a single-use system using disposable canisters to a central delivery system with reusable containers and dry concentrate powder significantly reduced the environmental impact of acid concentrate delivery at UMC Utrecht. Carbon emissions fell by 58% of the original value, and all three endpoint indicators (human health, ecosystem quality, and resource scarcity) were reduced by more than half.

The strong environmental performance of the central delivery system highlights its potential for broader global adoption. Even with longer transport to Italy, Kenya, and the Philippines, the centralized system showed lower overall impacts due to reduced packaging and weight. Although overseas shipping raised emissions in categories such as terrestrial acidification and particulate formation, the environmental gains from container reuse and streamlined delivery remain substantial. These results emphasize the critical role of reuse in mitigating impacts from plastic production and end-of-life treatment, even when return shipping is required.

Widespread adoption of central delivery systems could significantly enhance the sustainability of dialysis centers, particularly in high income countries. In resource-limited settings additional challenges must be considered, including higher upfront investment, limited access to technical support, and increased vulnerability to equipment failure and maintenance issues. Nevertheless, with further investment and the development of sustainable business models, centralized systems have the potential to advance both environmental and healthcare capacity globally.

Further policies accelerating the adoption of sustainable technologies, such as central concentrate delivery systems, will be essential to achieving international sustainability goals, including the European Green Deal. This will require coordinated action: policy makers can provide incentives and investment, healthcare providers can incorporate sustainability criteria in procurement, and manufacturers can continue to innovate in product design.

## Supplementary Information

Below is the link to the electronic supplementary material.

Supplementary Material 1

Supplementary Material 2

## Data Availability

All data generated or analyzed during this study are included in this published article and its supplementary material.

## Abbreviations

CCDS: Central Concentrate Delivery System
CKD: Chronic Kidney Disease
DALYs: Disability-Adjusted Life Years
HD: Hemodialysis
HDPE: High-Density Polyethylene
ISO: International Organization for Standardization
KRT: Kidney Replacement Therapy
LCA: Life Cycle Assessment
LCIA: Life Cycle Impact Assessment
LMICs: Low-and Middle-Income Countries
RO: Reverse Osmosis
UMC Utrecht: University Medical Center Utrecht

## Life cycle assessment impact categories

TA: Acidification: Terrestrial
GWP: Global Warming Potential
FEc: Ecotoxicity: Freshwater
MEc: Ecotoxicity: Marine
TEc: Ecotoxicity: Terrestrial
FR: Fossil Energy Resources
FEu: Eutrophication: Freshwater
Meu: Eutrophication: Marine
HCT: Human toxicity: Carcinogenic
HNCT: Human toxicity: Non-Carcinogenic
IR: Ionizing Radiation
LU: Land Use
MR: Mineral Resources
OD: Ozone Depletion
FP: Particulate Matter Formation
OF: Photochemical Oxidant Formation
WC: Water Consumption
EQ: Ecosystem Quality
HH: Human Health
RS: Resource Scarcity
