# Supplementary material for: Sustainable and accessible hemodialysis: life cycle assessment on central acid delivery system
Source: BMC Nephrol. 2025 Nov 5;26:619. doi: 10.1186/s12882-025-04499-0 (PMC12587493; doi:10.1186/s12882-025-04499-0)
Supplement: Supplementary file 1 — Supplementary Material 1 [file 12882_2025_4499_MOESM1_ESM.docx]

# Supplementary Methods: Inventory Analysis

## Production of acid concentrate

The raw materials and resources involved in the production processes of both the canister and central concentrate delivery systems are summarized in Table S1. Quantities for each system are presented per canister or per barrel, with values calculated relative to the functional unit, defined as 624 L of acid concentrate.

The composition of raw chemicals was obtained from the product labels. Both concentrates are designed to be diluted to the same dialysate composition (K+: 2 mmol/L, Ca²⁺: 1.25 mmol/L, Glucose: 1 g/L). Following a change in manufacturer, slight differences were noted in the sodium concentration (Dirinco: 4,500 mmol/L; B. Braun: 4,635 mmol/L) and in the form of glucose used (Dirinco: anhydrous; B. Braun: monohydrate) in the acid concentrate. These differences contribute to a minor variation in total product weight. However, the difference is minimal and therefore not expected to contribute meaningfully to the overall LCA results.

Data regarding electricity and water consumption for concentrate production in the central concentrate delivery system were provided by the manufacturer. For the canister-based system, energy consumption was assumed to be equivalent to that of the centralized system, taking into consideration that both systems employ comparable production processes.

In the central concentrate delivery system, an additional electricity input is included to account for the energy required to produce the dry concentrate. This value is estimated by proportionally scaling the total energy consumption according to the weight of material handled (216.4 kg of dry concentrate compared to 622 L of acid concentrate solution, as shown in Table 1). The total weight of one batch of acid concentrate (622 L) is calculated by summing the weight of 530 L of purified water used in formulation and 216.4 kg of dry concentrate.

| Table S1: Inventory of the production process of acid concentrate. | | | | | | | |
| --- | --- | --- | --- | --- | --- | --- | --- |
| **Canister** | | | | **Central Concentrate Delivery** | | | |
| **Item** | **Quantity** | | **Unit** | **Item** | **Quantity** | | **Unit** |
|  | *per canister (6L)* | *per FU* |  |  | *per barrel*  *(216.4 kg)* | *per FU* |  |
| **Raw Chemicals** | | | | **Raw Chemicals** | | | |
| NaCl | 1.58 | 164.11 | kg | NaCl | 168.49 | 169.03 | kg |
| KCl | 0.04 | 4.19 | kg | KCl | 4.17 | 4.19 | kg |
| CaCl_2_·2H_2_O ^a^ | 0.05 | 5.16 | kg | CaCl_2_·2H_2_O ^a^ | 5.14 | 5.16 | kg |
| MgCl_2_·6H_2_O ^b^ | 0.03 | 2.85 | kg | MgCl_2_·6H_2_O ^b^ | 2.85 | 2.85 | kg |
| Acetic Acid | 0.05 | 5.06 | kg | Acetic Acid | 5.04 | 5.06 | kg |
| Glucose (anhydrous) | 0.27 | 28.08 | kg | Glucose (monohydrate) ^c^ | 30.71 | 30.81 | kg |
|  |  |  |  |  |  |  |  |
| **Concentrate Production** | | | | **Dry Concentrate Production (Manufacturer)** | | | |
| Electricity ^d^ | - | 3.8 | kWh | Electricity ^e^ | 1.1 | 1.1 | kWh |
| Purified Water | 5.20 | 539 | kg |  |  |  |  |
|  |  |  |  | **Concentrate Production (Hospital)** | | | |
|  |  |  |  | Electricity | 3.8 | 3.8 | kWh |
|  |  |  |  | Purified Water ^f^ | 590 | 592 | kg |
| 1. 1kg of CaCl_2_·2H_2_O is modelled as 0.7545 kg of CaCl_2_ and 0.2455 kg of H_2_O. 2. 1kg of MgCl_2_·6H_2_O is modelled as 0.4684 kg of CaCl_2_ and 0.5316 kg of H_2_O. 3. 1kg of Glucose (monohydrate) is modelled as 0.91 kg of Glucose (anhydrate) and 0.09 kg of H_2_O. 4. Electricity consumption for concentrate production in the canister system is assumed to be equivalent to Central Concentrate Delivery. 5. The electricity consumption for filling the dry concentrate is the scaled proportionally to the weight of material processed. 6. Includes 530 L of purified water for solution formulation and 60 L for equipment rinsing after each batch.   FU = functional unit | | | | | | | |

## Packaging and transportation

The materials used for packaging and the total transported weight of the products are summarized in Table S2. Both the canisters and barrels are produced using blow molding, while the caps are manufactured through injection molding, with high density polyethylene (HDPE) as the primary material. Market data were used to model these manufacturing processes.

The total transported weight includes the acid concentrate (in either solution or dry form), the packaging materials (disposable canisters or reusable barrels), and the transport carriers (wooden pallets or steel carts). Both products are manufactured in Germany and are assumed to be transported directly to the delivery sites by lorry. For overseas shipments, container ships are used, with the shortest available route selected.

Regarding transport carriers, 60 single-use canisters are loaded onto Euro 1 wooden pallets, whereas reusable barrels are distributed using steel carts, which are continuously reused. Additionally, packaging film made from linear low density polyethylene (LLDPE) is used to secure and protect the products during transportation.

Transport distances for the different delivery locations are provided in Table S3. In all cases, refrigerated transport (reefer containers) was assumed, due to storage temperature requirements specified in the datasheets of the concentrates [1, 2]. Distances are estimated with Google Maps [3] and Sea Distances [4].Transport-related energy consumption is calculated by multiplying the total transported weight by the travel distance, expressed in ton kilometers (t·km). The resulting values are presented in Table S4.

| Table S2: Inventory of packaging and transportation of acid concentrates. | | | | | | | |
| --- | --- | --- | --- | --- | --- | --- | --- |
| **Canister** | | | | **Central Concentrate Delivery** | | | |
| **Item** | **Quantity** | | **Unit** | **Item** | **Quantity** | | **Unit** |
|  | *per canister (6L)* | *per FU* |  |  | *per barrel (216.4 kg)* | *per FU* |  |
| **Delivery** |  |  |  | **Delivery** |  |  |  |
| Canister, HDPE | 0.26 | 27.04 | kg | Barrel, HDPE ^a^ | 18.00 | 18.05 | kg |
| Cap, HDPE | 0.01 | 1.04 | kg | Caps, HDPE | 0.29 | 0.29 | kg |
| Film, LLDPE | 0.002 | 0.21 | kg | Film, LLDPE | 0.12 | 0.12 | kg |
| Wooden Pallet ^b^ | 0.42 | 43.68 | kg | Steel Cart ^c^ | 14.80 | 14.85 | kg |
| Concentrate | 7.20 | 748.80 | kg | Dry Concentrate | 216.40 | 217.10 | kg |
| Total Weight | 7.90 | 821.60 | kg | Total Weight | 249.60 | 250.40 | kg |
|  |  |  |  |  | | | |
|  |  |  |  | **Return of empty container** | | | |
|  |  |  |  | Steel Cart | 14.80 | 14.85 | kg |
|  |  |  |  | Container | 18.29 | 18.35 | kg |
|  |  |  |  | Total Weight | 33.10 | 33.20 | kg |
| 1. The value shown is the considered weight for transport. In packaging and end-of-life treatments, the value is divided by the number of use cycles for further calculation. 2. 60 canisters are transported per euro pallet (25kg). 3. Steel carts are not disposed, therefore although considered for the weight in transport it is not considered for packaging and end-of-life treatments.   FU = functional unit; HDPE = high density polyethylene; LLDPE = linear low density polyethylene. | | | | | | | |

| Table S3: Transport distance for acid concentrate delivery under different system scenarios.  For UMC Utrecht (Netherlands), both canister and central concentrate delivery systems are included. The international sites including University of Modena and Reggio Emilia (Italy), Kenyatta National Hospital (Nairobi, Kenya), and Philippine General Hospital (Manila, Philippines) are evaluated using the central system. | | | | | |
| --- | --- | --- | --- | --- | --- |
| **System** | **Destination** | **Road:**  **Factory to Port (km)** | **Sea:**  **Port to Port (km)** | **Road:**  **Port to Hospital (km)** | **Total Distance (km)** |
| Canister | Utrecht, NL | 710 (direct) | - | - | 710 |
| Central | Utrecht, NL | 245 (direct) | - | - | 245 |
| Central | Modena, IT | 1,300  (direct) | - | - | 1,300 |
| Central | Nairobi, KE | 120 (to Bremerhaven) | 12,000 (via Mombasa Port) | 480 (to Kenyatta Hospital) | 12,600 |
| Central | Manila, PH | 120 (to Bremerhaven) | 18,200 (via Manila Port) | 4 (to Philippine General) | 18,324 |

| Table S4: Transport need per functional unit for different delivery sites. | | | | | | |
| --- | --- | --- | --- | --- | --- | --- |
| **System Model** | **Scenario Destination** | **Direction** | **Road:**  **Mfg. to Port  (t⋅km)** | **Sea:**  **Port to Port (t⋅km)** | **Road:**  **Port to Hospital (t⋅km)** | **Total Transport**  **(t⋅km)** |
| **Canister** | Utrecht, NL | Distance (km) | 710 | - | - |  |
|  |  | One-way | 583 | - | - | **583** |
| **Central Concentrate Delivery** | Utrecht, NL | Distance (km) | 245 | - | - |  |
|  |  | Outbound | 61 | - | - |  |
|  |  | Return | 8 | - | - | **70** |
|  | Modena, IT | Distance (km) | 1,300 | - | - |  |
|  |  | Outbound | 326 | - | - |  |
|  |  | Return | 43 | - | - | **369** |
|  | Nairobi, KE | Distance (km) | 120 | 12,000 | 480 |  |
|  |  | Outbound | 30 | 3,005 | 120 |  |
|  |  | Return | 4 | 398 | 16 | **3,573** |
|  | Manila, PH | Distance (km) | 120 | 18,200 | 4 |  |
|  |  | Outbound | 30 | 4,557 | 1 |  |
|  |  | Return | 4 | 604 | 0 | **5,197** |
| NL = Netherlands; IT = Italy; KE = Kenya; PH = Philippines. | | | | | | |

## End-of-life

The waste generated after use is summarized in Table S5. Wastewater resulting from the dialysis process is assumed to be discharged directly into the municipal sewage system. The single-use canisters are discarded after each use, whereas the reusable barrels are assumed to undergo 15 usage cycles. Caps and packaging films are disposed of after every use cycle. Wooden pallets are assumed to be reused for 20 transport cycles before disposal.

All solid waste, including plastics and wooden pallets, is assumed to enter recycling streams. The transport of this waste to recycling or treatment facilities has been excluded from the analysis, as the distances involved are short and the weight are relatively low compared to the transport of filled acid concentrate canisters/barrels from the manufacturer to the hospital. Consequently, its contribution to the overall environmental impact is anticipated to be minimal.

| Table S5: Inventory for end-of-life treatment per functional unit. | | | |
| --- | --- | --- | --- |
| **Item** | **Canister** | **Central Concentrate Delivery** | **Unit** |
| Wastewater | 624 | 624 | L |
| Plastic, HDPE | 28.1 | 1.5 | kg |
| Plastic, LLDPE | 0.2 | 0.1 | kg |
| Wood | 2.2 | - | kg |

## References

[1] M. T. N. N. GmbH, "MSDS: Zuurconcentraat voor bicarbonaatdialyse, A-component en Citrasate®," MTN Neubrandenburg GmbH, 2023. [Online]. Available: [Internal document, not publicly available]

[2] M. Intermedt and H. Technik Gmb, "Instructions for Use: ECOCart," Intermedt Medizin & Technik GmbH, 2024. [Online]. Available: <https://www.intermedt.de/wp-content/uploads/IFU-ECOCart-2024-07.pdf>

[3] Google LLC. "Google Maps." <https://www.google.com/maps> (accessed May 25, 2025).

[4] Sea-Distances.org. "Sea Distances - Voyage Planning." <https://sea-distances.org> (accessed March 20, 2025).
