# Supplementary material for: Sustainable and accessible hemodialysis: life cycle assessment on central acid delivery system
Source: BMC Nephrol. 2025 Nov 5;26:619. doi: 10.1186/s12882-025-04499-0 (PMC12587493; doi:10.1186/s12882-025-04499-0)
Supplement: Supplementary file 2 — Supplementary Material 2 [file 12882_2025_4499_MOESM2_ESM.docx]

Supplementary Results: Life Cycle Assessment Results of the Canister and Central Concentrate Delivery Systems

| **Table S6: Global Warming Potential (carbon footprint) contributions by of canister and central delivery system for different scenario (kg CO₂-Eq).** Results are presented across four country settings: the Netherlands (NL), Italy (IT), Kenya (KE), and the Philippines (PH). | | | | | | |
| --- | --- | --- | --- | --- | --- | --- |
| **Scenario** | **Acid Production** | **Packaging** | **Land Transport** | **Sea**  **Transport** | **Waste Management** | **Total** |
| Canister (NL) | 112.78 | 105.26 | 83.41 | 0.00 | 11.22 | 312.67 |
| Central (NL) | 114.75 | 6.89 | 9.94 | 0.00 | 0.86 | 132.44 |
| Central (IT) | 114.64 | 6.89 | 52.72 | 0.00 | 0.86 | 175.11 |
| Central (KE) | 114.46 | 6.89 | 24.33 | 77.81 | 0.68 | 224.17 |
| Central (PH) | 117.52 | 6.89 | 5.03 | 118.01 | 0.68 | 248.13 |

Table S7: Comparison of annual life cycle impact results for canister-based and central concentrate delivery systems in a 60-patient dialysis unit. Results are presented across four country settings: the Netherlands (NL), Italy (IT), Kenya (KE), and the Philippines (PH). All values are internally normalized to the single-patient system in the Netherlands (Canister NL = 100%).

| **Impact category** | **Abbrev.** | **Reference unit** | **Single (NL)** | **Central (NL)** | | **Central (IT)** | | **Central (KE)** | | **Central (PH)** | |
| --- | --- | --- | --- | --- | --- | --- | --- | --- | --- | --- | --- |
|  |  |  | **Result** | **Result** | **Norm** | **Result** | **Norm** | **Result** | **Norm** | **Result** | **Norm** |
| Acidification: Terrestrial | TA | kg SO_2_-Eq | 1.08E+00 | 5.74E-01 | 53% | 6.98E-01 | 65% | 1.53E+00 | 142% | 1.95E+00 | 181% |
| Global Warming Potential | GWP | kg CO_2_-Eq | 3.13E+02 | 1.32E+02 | 42% | 1.75E+02 | 56% | 2.24E+02 | 72% | 2.48E+02 | 79% |
| Ecotoxicity: Freshwater | FEc | kg 1,4-DCB-Eq | 1.57E+01 | 1.10E+01 | 70% | 1.21E+01 | 77% | 1.32E+01 | 84% | 1.37E+01 | 87% |
| Ecotoxicity: Marine | MEc | kg 1,4-DCB-Eq | 2.15E+01 | 1.43E+01 | 67% | 1.63E+01 | 76% | 1.74E+01 | 81% | 1.79E+01 | 83% |
| Ecotoxicity: Terrestrial | TEc | kg 1,4-DCB-Eq | 3.02E+03 | 1.26E+03 | 42% | 2.13E+03 | 70% | 1.73E+03 | 57% | 1.43E+03 | 47% |
| Fossil Energy Resources | FR | kg oil-Eq | 1.17E+02 | 3.77E+01 | 32% | 5.15E+01 | 44% | 6.40E+01 | 55% | 6.98E+01 | 60% |
| Eutrophication: Freshwater | FEu | kg P-Eq | 1.11E-01 | 8.04E-02 | 73% | 8.37E-02 | 76% | 8.66E-02 | 78% | 8.94E-02 | 81% |
| Eutrophication: Marine | MEu | kg N-Eq | 5.25E-02 | 4.57E-02 | 87% | 4.70E-02 | 89% | 4.95E-02 | 94% | 5.02E-02 | 96% |
| Human toxicity: Carcinogenic | HCT | kg 1,4-DCB-Eq | 2.11E+01 | 1.17E+01 | 55% | 1.38E+01 | 65% | 1.65E+01 | 78% | 1.80E+01 | 85% |
| Human toxicity: Non-Carcinogenic | HNCT | kg 1,4-DCB-Eq | 3.33E+02 | 2.07E+02 | 62% | 2.40E+02 | 72% | 2.40E+02 | 72% | 2.40E+02 | 72% |
| Ionizing Radiation | IR | kBq Co-60-Eq | 1.71E+01 | 9.15E+00 | 53% | 9.59E+00 | 56% | 9.68E+00 | 56% | 9.22E+00 | 54% |
| Land Use | LU | m^2^·a crop-Eq | 2.89E+01 | 1.67E+01 | 58% | 1.89E+01 | 65% | 1.77E+01 | 61% | 1.69E+01 | 59% |
| Mineral Resources | MR | kg Cu-Eq | 3.65E+01 | 3.50E+01 | 96% | 3.58E+01 | 98% | 3.63E+01 | 100% | 3.65E+01 | 100% |
| Ozone Depletion | OD | kg CFC-11-Eq | 2.90E-04 | 2.47E-04 | 85% | 2.58E-04 | 89% | 2.96E-04 | 102% | 3.15E-04 | 109% |
| Particulate Matter Formation | FP | kg PM2.5-Eq | 4.72E-01 | 2.37E-01 | 50% | 2.95E-01 | 62% | 6.16E-01 | 130% | 7.87E-01 | 167% |
| Photochemical Oxidant Formation | OF | kg NO_x_-Eq | 2.13E+00 | 8.31E-01 | 39% | 1.30E+00 | 61% | 3.48E+00 | 164% | 4.58E+00 | 215% |
| Water Consumption | WC | m^3^ | 5.54E+00 | 4.53E+00 | 82% | 4.63E+00 | 84% | 4.64E+00 | 84% | 4.65E+00 | 84% |
| Ecosystem Quality | EQ | species.yr | 1.62E-06 | 7.73E-07 | 48% | 9.82E-07 | 61% | 1.43E-06 | 88% | 1.64E-06 | 101% |
| Human Health | HH | DALYs | 7.46E-04 | 3.68E-04 | 49% | 4.59E-04 | 62% | 7.16E-04 | 96% | 8.52E-04 | 114% |
| Resource Scarcity | RS | USD 2013 | 5.00E+01 | 1.96E+01 | 39% | 2.56E+01 | 51% | 3.12E+01 | 62% | 3.35E+01 | 67% |

Table S8: Comparison of sensitivity results for canister system and central concentrate delivery. The scenario of delivery to the Netherlands was used for the canister system to represent local delivery, while delivery to Kenya was used for the central concentrate delivery system to represent long-distance overseas delivery.

| **Process** | **Adjusted Parameter** | **Canister System (delivered to Netherlands)** | | **Central Delivery System (delivered to Kenya)** | |
| --- | --- | --- | --- | --- | --- |
|  |  | **Value Changed** | **Change in Human Health Impact (DALYs)** | **Value Changed** | **Change in Human Health Impact (DALYs)** |
| Production | Water and energy used + 100% | 3.8 kWh 🡪 7.6 kWh  539 kg 🡪 1078 kg | 1.2% | 4.9 kWh 🡪 9.8 kWh  592 kg 🡪 1184 kg | 2.0% |
| Packaging | Reused 🡪 Not reused | 1 cycle 🡪 1 cycle | 0.0% | 15 cycles 🡪 1 cycle | 18.5% |
| Transportation | Distance +20% | 245 km 🡪 294 km | 5.1% | 12,600 km 🡪 15,120 km | 10.0% |
| End-of-life | Recycled 🡪 Incinerated | - | 5.3% | - | 0.4% |
| Combined | All mentioned above | - | 11.5% | - | 35.1% |
